# Supplementary material for: Pharmacological polyamine catabolism upregulation with methionine salvage pathway inhibition as an effective prostate cancer therapy
Source: Nat Commun. 2020 Jan 7;11:52. doi: 10.1038/s41467-019-13950-4 (PMC6946658; doi:10.1038/s41467-019-13950-4)
Supplement: Supplementary file 1 — Supplementary Information [file 41467_2019_13950_MOESM1_ESM.pdf]

Pharmacological Polyamine Catabolism upregulation with Methionine Salvage Pathway inhibition as an effective Prostate Cancer therapy

Affronti et al.

Supplementary Materials:

Supplementary Figure legends

Supplementary Fig.1. Flow Cytometry for LNCAP, CWR22Rv1, and C42 cells.

Supplementary Fig.2. Intracellular BENSpm concentration and enzymatic activity of ODC1 and AMD1.

Supplementary Fig.3 SMOX Knockdown rescues ROS in LNCaP cells.

Supplementary Fig.4 TXNRD2 over expression affects response to hydrogen peroxide.

Supplementary Fig.5. Long term toxicity analysis of MTDIA + BENSpm.

Supplementary Fig.6. 8-oxo-dG staining of CWR22Rv1 xenografts.

Supplementary Fig.7. Comparison of BENSpm dosing schedules.

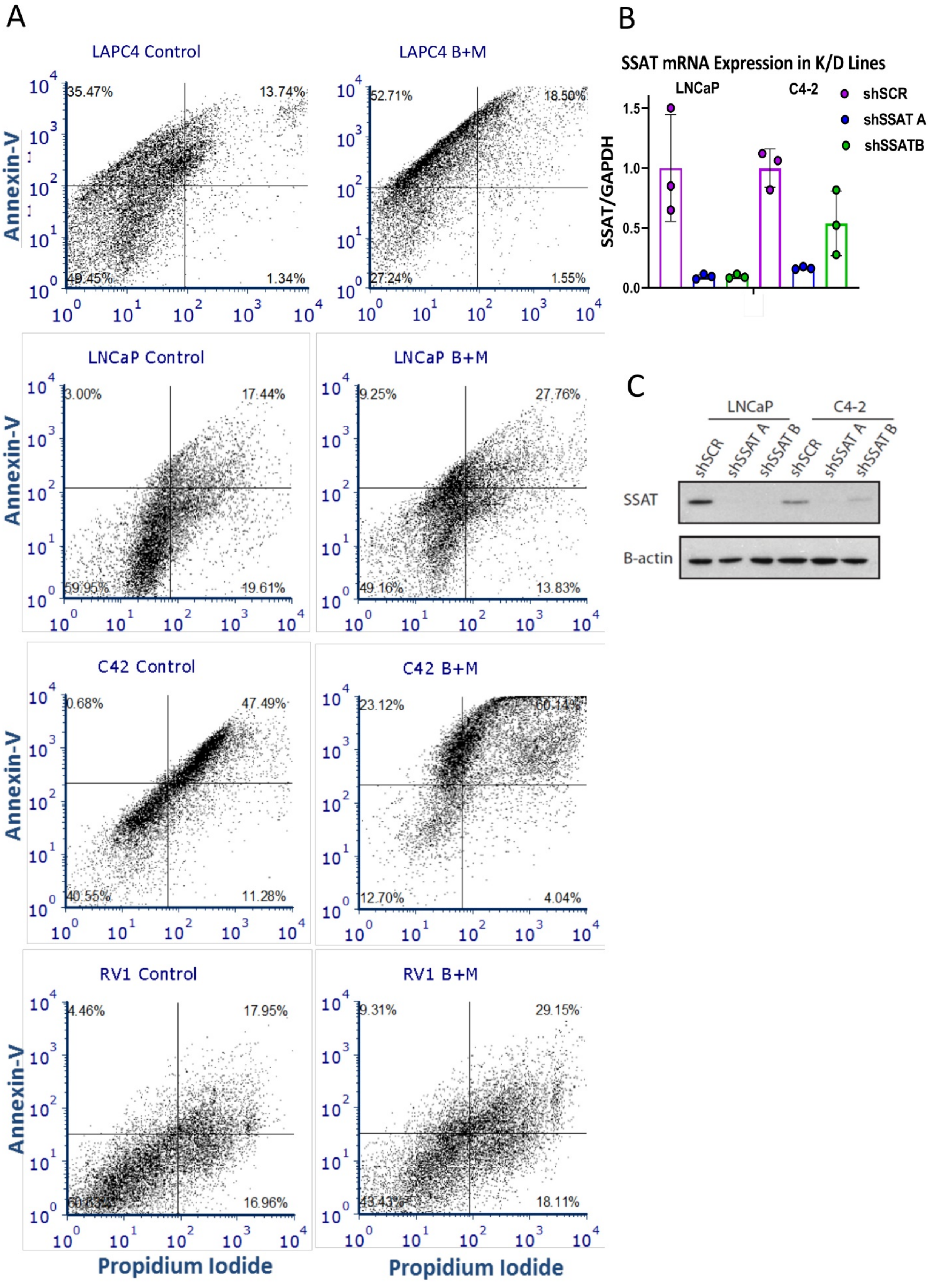
A
LAPC4 Control
35.47%
13.74%
49.45%
1.34%
LAPC4 B+M
52.71%
18.50%
27.24%
1.55%
LNCaP Control
3.00%
17.44%
59.95%
19.61%
LNCaP B+M
9.25%
27.76%
49.16%
13.83%
C42 Control
0.68%
47.49%
40.55%
11.28%
C42 B+M
23.12%
12.70%
4.04%
RV1 Control
4.46%
17.95%
16.96%
RV1 B+M
9.31%
29.15%
43.43%
18.11%
Annexin-V
Propidium Iodide
B
SSAT mRNA Expression in K/D Lines
LNCaP
C4-2
shSCR
shSSAT A
shSSATB
SSAT/GAPDH
1.5
1.0
0.5
0.0
C
LNCaP
C4-2
shSCR
shSSAT A
shSSAT B
shSCR
shSSAT A
shSSAT B
SSAT
B-actin

Supplementary Figure 1: (A) LNCaP, LAPC4, C4-2 and CWR22Rv1 cells treated with vehicle control or 1 nM MTDIA and 1 µM BENSpm in the presence of 20 µM MTA for 8 days, were analyzed by Flow Cytometry following staining with Annexin-V and Propidium iodide. Scatterplots depict cell populations stained with Annexin-5 (indicative of apoptosis) and/or Propidium Iodide (indicative of necrotic cells). **(B)** *SSAT* RNA expression measured by Real-Time rt-PCR in LNCaP and C4-2 cells transfected with a scramble control shRNA (shSCR-black bars), or one of two shRNAs to *SSAT* (shSSAT A (light gray bars) or shSSAT B (dark gray bars), n=3). Error bars represent standard deviation of the mean. **(C)** Protein expression measured by western blot for LNCaP and C4-2 cells containing shSCR, sh*SSAT* A or sh*SSAT* B. Cells were treated for 96 hours with 2.5 µM BENSpm to increase detectable SSAT protein expression.

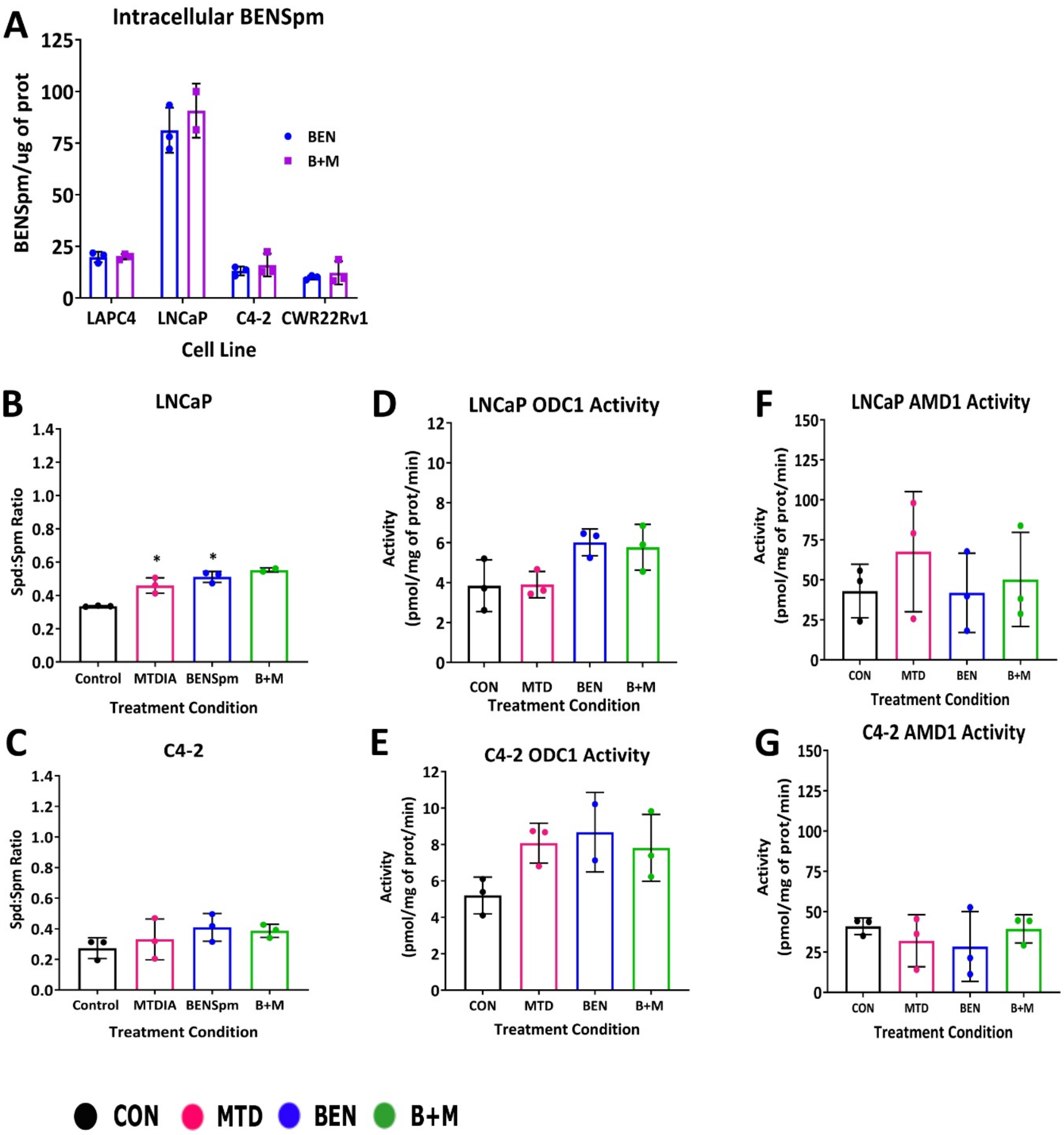

Supplementary Figure 2: (A) LAPC4, LNCaP, C4-2, and CWR22RV1 cells were treated for 8 days with either 1 μM BENSpm (Blue dotted bars) or the combination (B+1 nM MTDIA). Intracellular BENSpm was measured by UPLC. Cells were treated with control (black bars), 1 nM MTDIA (dotted bars), 1 μM BENSpm (striped bars), or the combination (B+M) (white bars) in 20 μM MTA for 8 days. The spermidine (Spd) to spermine (Spm) synthase ratio as measured by UPLC in **(B)** LNCaP and **(C)** C4-2, indicative of polyamine biosynthetic flux. ODC1 enzyme activity assayed in **(D)** LNCaP and **(E)** C4-2. AMD1 enzyme

activity assayed in **(F)** LNCaP and **(G)** C4-2. Indicated specific enzymatic activity is reported as pmol of radiolabeled acetyl-CoA produced per minute relative to protein concentration (pmol/minute/mg of protein). Results for biological triplicates are shown. Statistical analyses were performed using an unpaired student t-test with Welch's correction. All values are compared to vehicle control. Error bars represent standard deviation of the mean.*:$p<0.05$

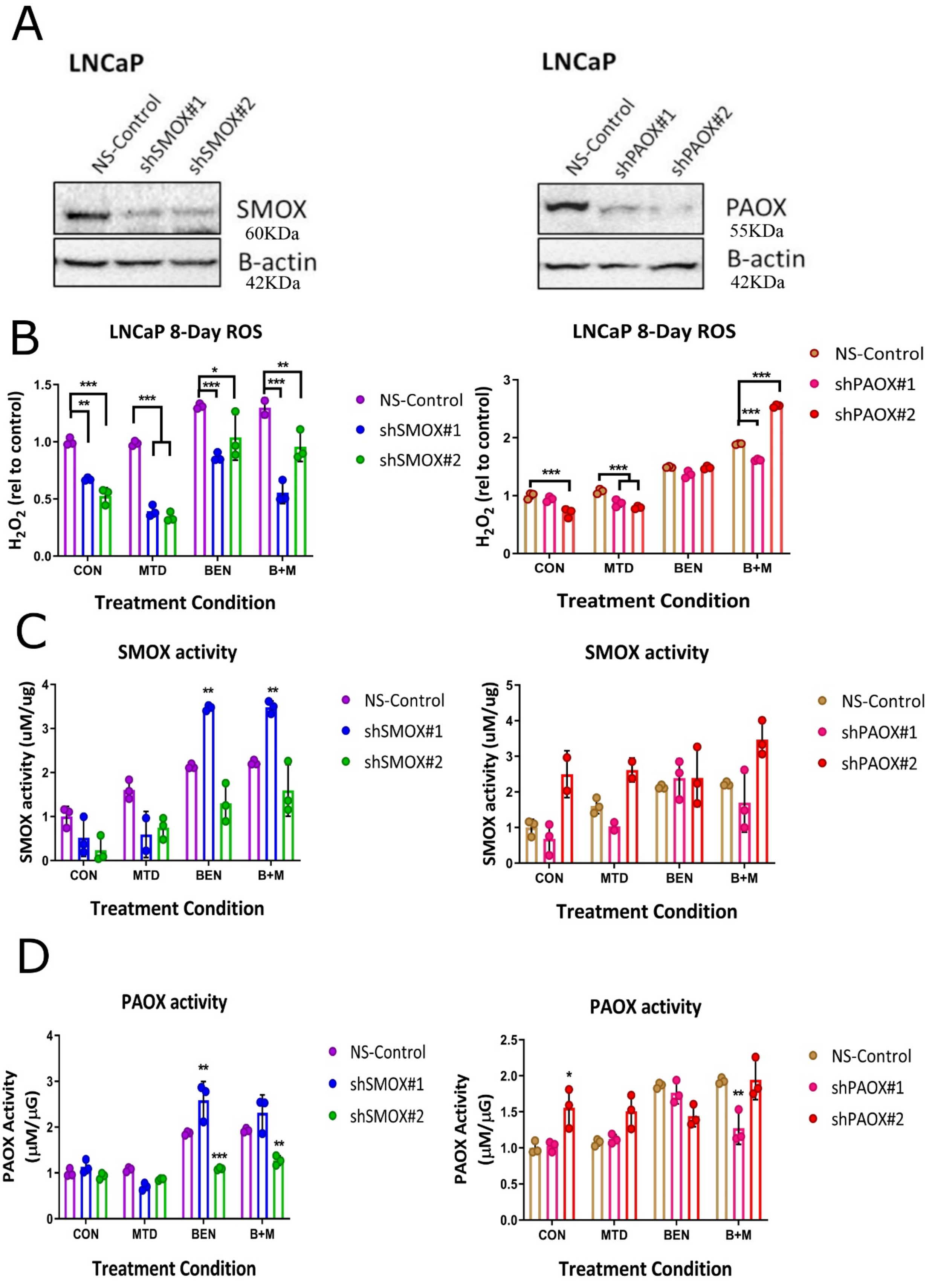
A
LNCaP
NS-Control
shSMOX#1
shSMOX#2
SMOX
60KDa
B-actin
42KDa
LNCaP
NS-Control
shPAOX#1
shPAOX#2
PAOX
55KDa
B-actin
42KDa
B
LNCaP 8-Day ROS
H2O2 (rel to control)
CON
MTD
BEN
B+M
Treatment Condition
NS-Control
shSMOX#1
shSMOX#2
LNCaP 8-Day ROS
H2O2 (rel to control)
CON
MTD
BEN
B+M
Treatment Condition
NS-Control
shPAOX#1
shPAOX#2
C
SMOX activity
SMOX activity (uM/ug)
CON
MTD
BEN
B+M
Treatment Condition
NS-Control
shSMOX#1
shSMOX#2
SMOX activity
SMOX activity (uM/ug)
CON
MTD
BEN
B+M
Treatment Condition
NS-Control
shPAOX#1
shPAOX#2
D
PAOX activity
PAOX Activity (μM/μG)
CON
MTD
BEN
B+M
Treatment Condition
NS-Control
shSMOX#1
shSMOX#2
PAOX activity
PAOX Activity (μM/μG)
CON
MTD
BEN
B+M
Treatment Condition
NS-Control
shPAOX#1
shPAOX#2

Supplementary Figure 3: (A) Western blot for antibodies against SMOX, PAOX, or β-Actin in LNCaP cells transfected with either a non-silencing control vector, or two different shRNAs for *SMOX* or *PAOX* target sequences. Cells were treated for 8 days with either vehicle control, 1nM MTDIA, 1μM BENSpm, or the combination (B+M) in the presence of 20μM MTA. **(B)** The relative reactive oxygen species (ROS) produced normalized to cell number (compared to non-silencing control cells treated with vehicle) in non-silencing, shSMOX, or shPAOX cells. **(C)** SMOX and (D) PAOX enzyme activity. Values are indicative of the amount of H2O2 produced normalized to protein concentration, relative to vehicle control treated non-silencing control cells. Results for biological triplicates are shown. Statistical analyses were performed using a two-way anova with multiple corrections. All comparisons are indicated by connecting lines. Error bars represent standard deviation of the mean *:$p<0.05$, **:$p<0.01$, ***:$P<0.001$.

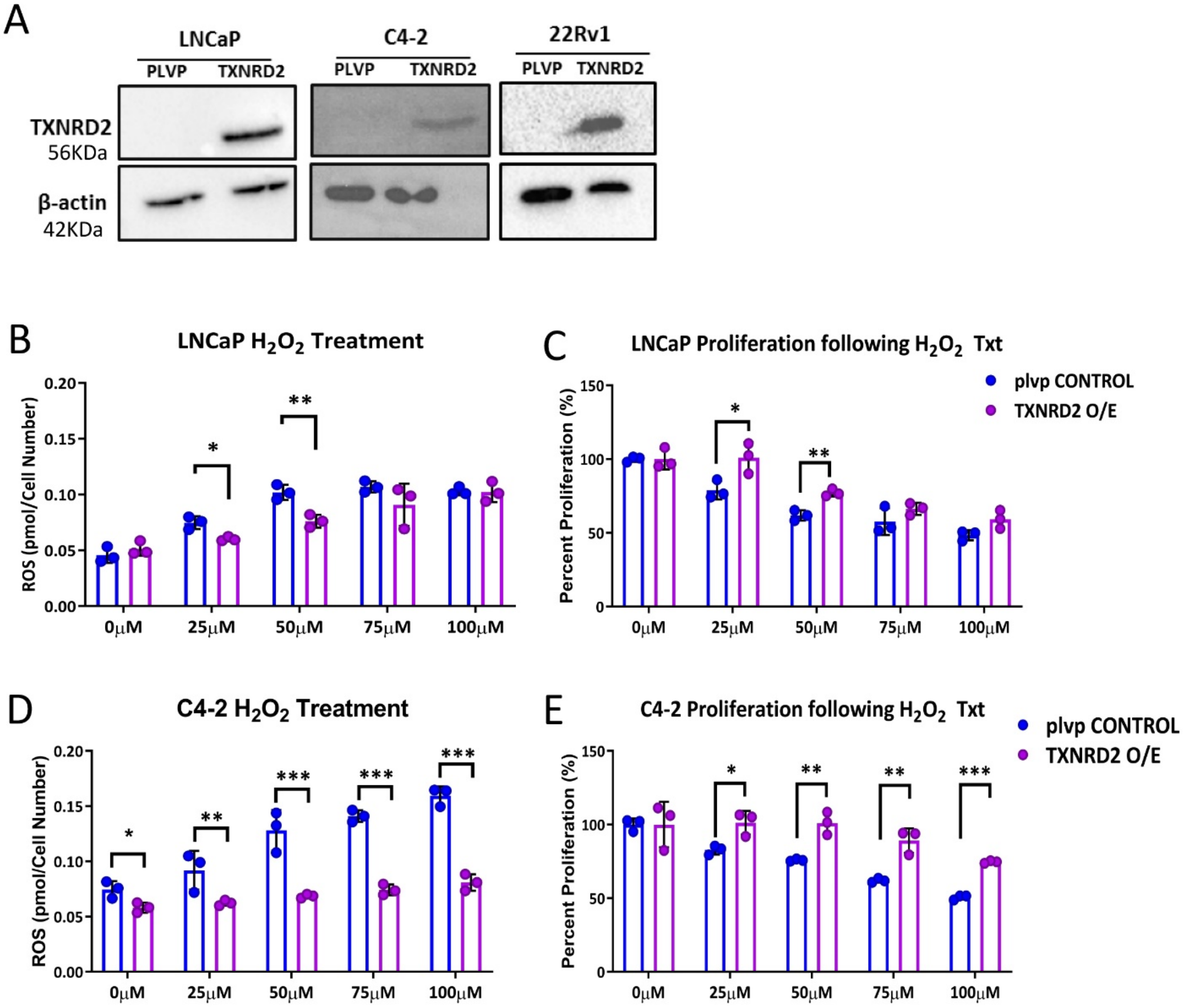

Supplementary Figure 4: (A) Western blot using antibodies against TXNRD2 and β-actin in cells transfected with PLVP vector control and *TXNRD2* overexpression construct. Cells were treated for 24 hours with 0, 25, 50, 75 and 100 μM H2O2 in the presence of 20 μM MTA. The amount of reactive oxygen species (ROS) produced normalized to cell number in cells containing vector control (PLVP) (black bars) or thioredoxin reductase 2 (*TXNRD2*) overexpression (O/E) (red bars) for **(B)** LNCaP and **(D)** C4-2. Percent cell proliferation (relative to vehicle control for each condition - PLVP or *TXNRD2* O/E) for **(C)** LNCaP and **(E)** C4-2. Results for biological triplicates are shown. Statistical analyses were performed using an unpaired student t-test with Welch's correction. All values are compared to vehicle control

unless otherwise indicated by connecting lines. Error bars represent standard deviation of the mean.*:p<0.05, **:p<0.01, ***:P<0.001.

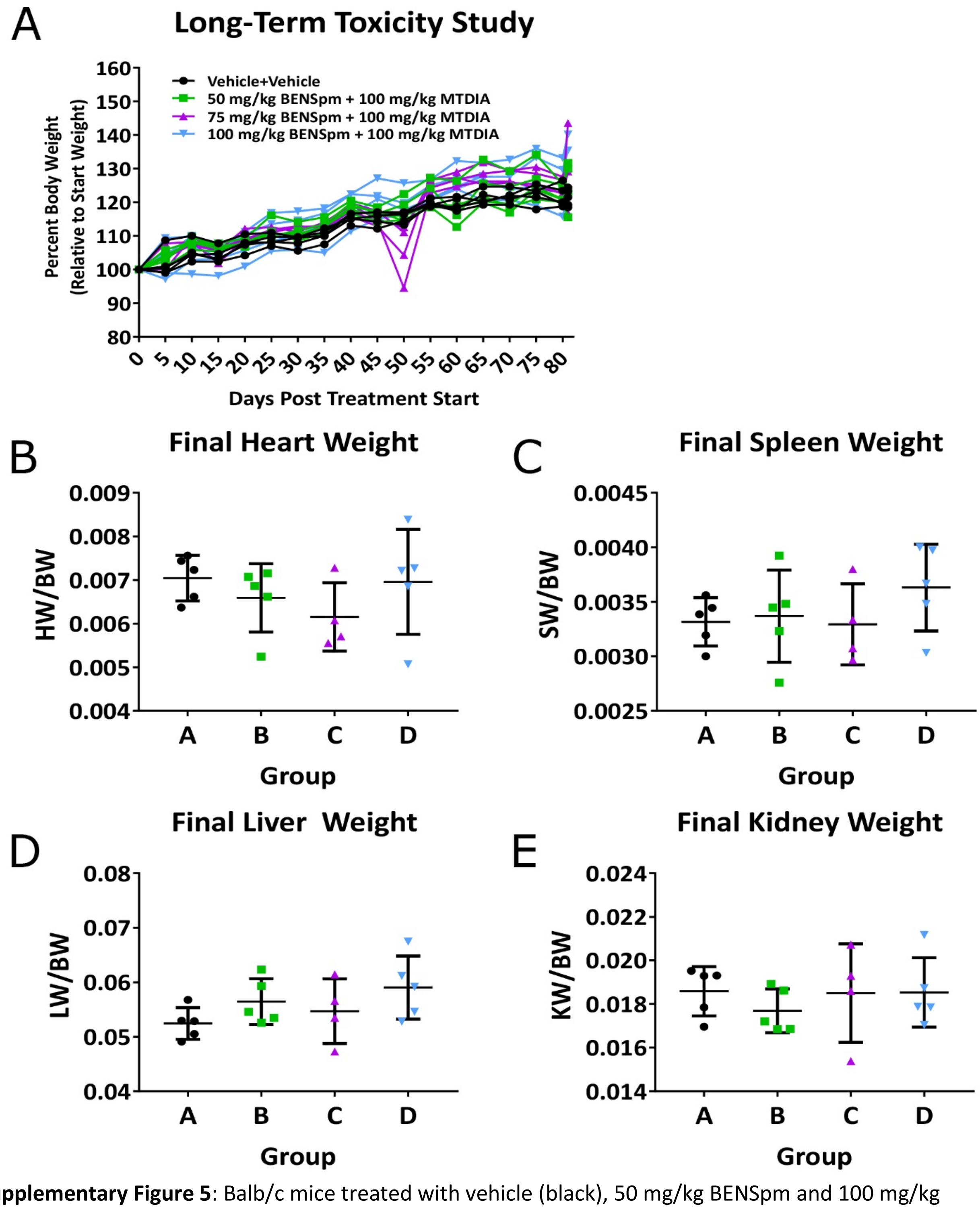

Supplementary Figure 5: Balb/c mice treated with vehicle (black), 50 mg/kg BENSpm and 100 mg/kg

MTDIA (green), 75 mg/kg BENSpm and 100 mg/kg MTDIA (purple), or 100 mg/kg BENSpm and 100 mg/kg MTDIA (blue) for three months. MTDIA was given continuously in the drinking water throughout the study. BENSpm was given as an i.p. injection once daily for 5 days, every 2 weeks. (A) Body weights measured for all animals normalized to the body weight at the study start date, day 0. The final (B) heart weight (HW), (C) spleen weight (SW), (D) liver weight (LW), and (E) kidney weight (KW) measured at sacrifice. For A-E, group A; n=5, group B; n=5, group C; n=4, and group D; n=5. Error bars represent standard deviation of the mean.

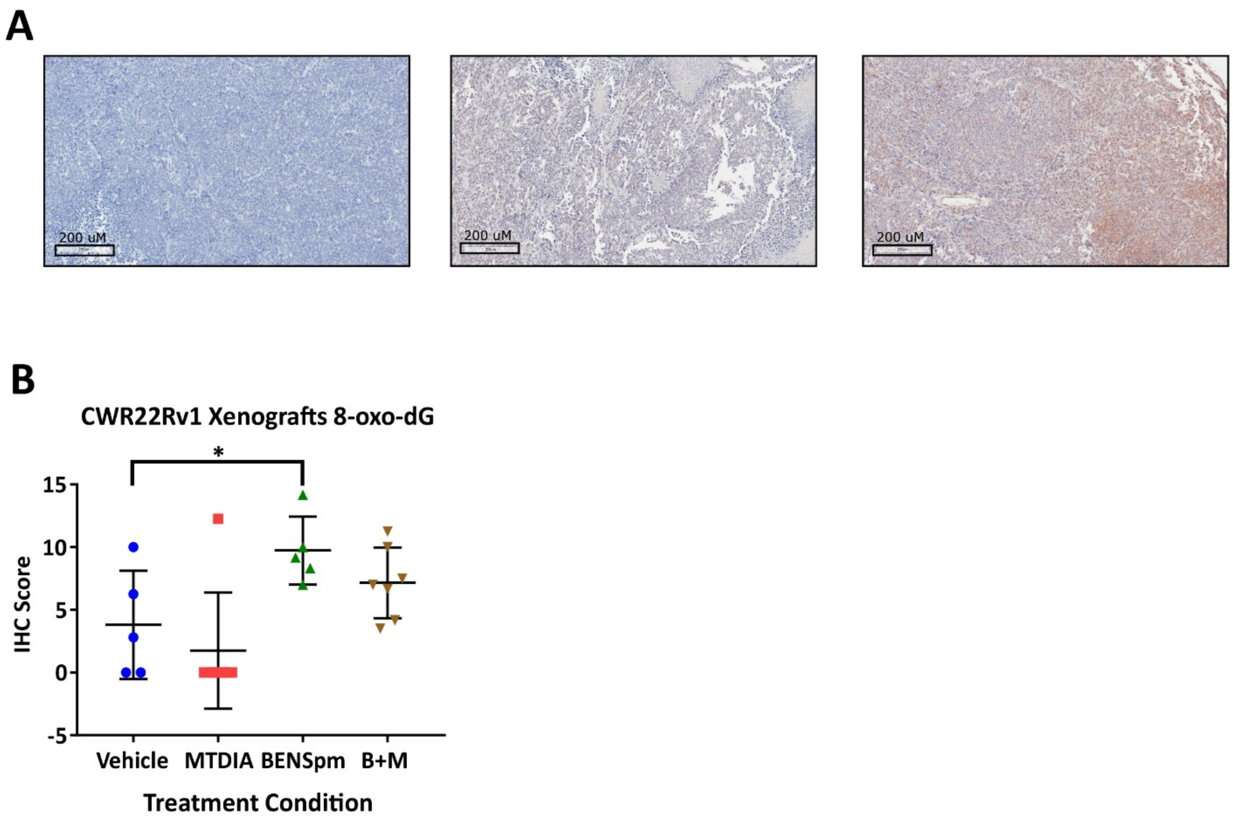

Supplementary Figure 6: (A) Images from 3 representative CWR22Rv1 tumors from immunohistochemical stained slides for 8-oxo-dG, a marker indicative of ROS induced DNA damage. Images are representative of tumors that stained low, medium and high for 8-oxo-dG. **(B)** IHC Scores for each tumor from the CWR22Rv1 xenograft study. IHC scores are representative of the average percent positive cells multiplied by the intensity of staining. Vehicle; n=5, MTDIA; n=7, BENSpm; n=7, and B+M;

n=7. Error bars represent standard deviation of the mean. Statistical analysis for IHC was performed using an unpaired student t-test with Welch's correction. *: p<0.05.

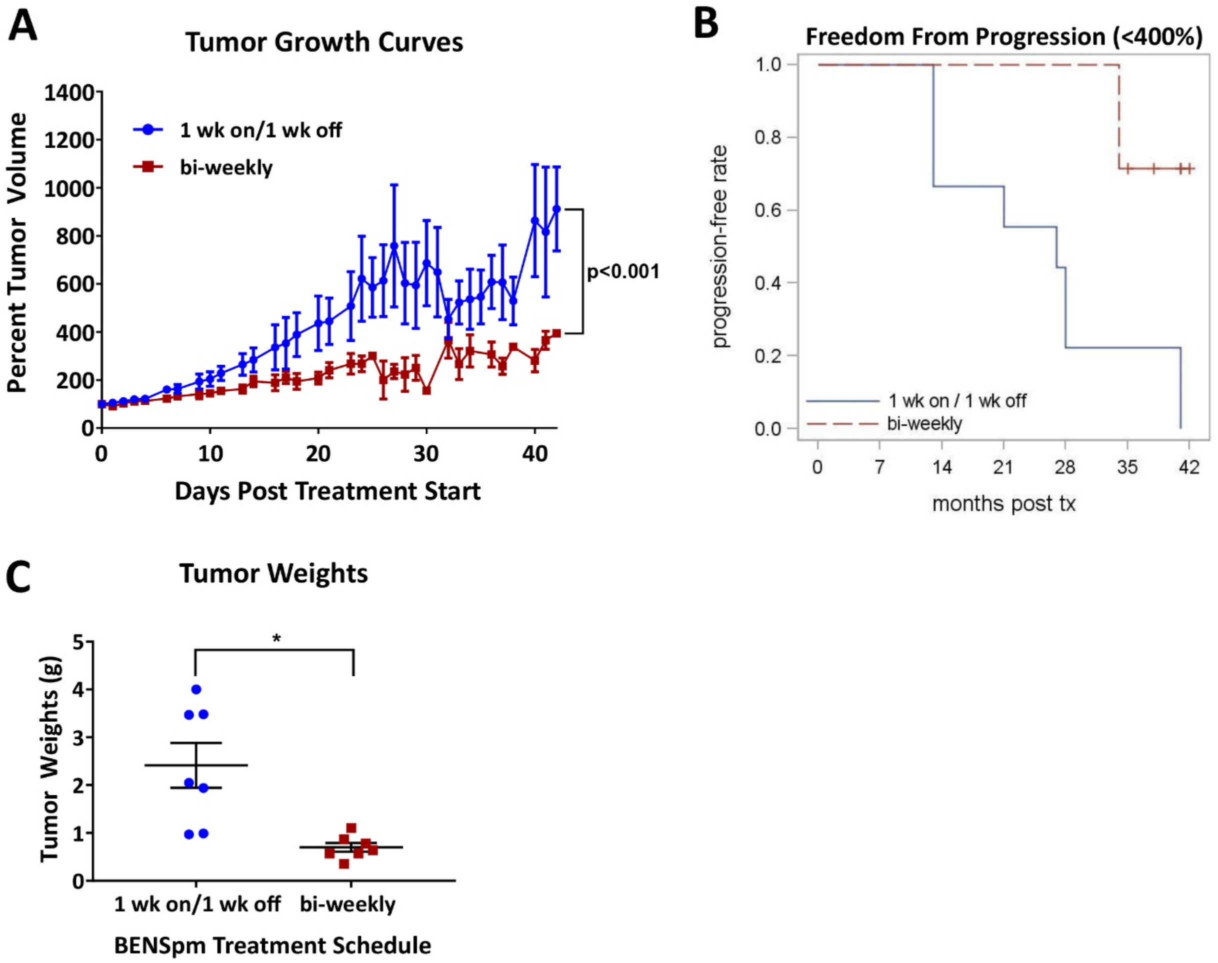

Supplementary Figure 7: Animals were subcutaneously implanted with $1x10^6$ CWR22Rv1 cells. Once tumors reached between 300-400 mm^3 animals were treated with 100 mg/kg BENSpm twice weekly (red) or 75 mg/kg BENSpm 1 time daily for 5 days (blue), repeated every two weeks to give a 1 week on/1 week off dosing schedule. All animals shown here are from the combination groups and therefore also received 50 mg/kg MTDIA in the drinking water continuously. **(A)** Mean tumor sizes over time are plotted for each animal of the treatment groups made relative to the starting tumor size at treatment day 0. The log relative tumor volume was modeled as a function of treatment, time, the time-treatment interaction, and random mouse effects and slopes using a linear mixed model. The growth rates were

compared using tests about the appropriate linear contrasts of model estimates (where 1 wk on/1 wk off; n=12, bi-weekly; n=11). **(B)** The graph indicates progression free survival where progression is defined as reaching 400%. **(C)** The tumor weights at sacrifice for each group. Error bars represent standard error of the mean (where 1 wk on/1 wk off; n=12, bi-weekly; n=11). Statistical analysis for tumor weights was performed using an unpaired student t-test with Welch's correction. *: $p<0.05$.
